# Supplementary material for: Pre-Diagnostic Saliva Microbiota of School-Aged Children Who Developed Type 1 Diabetes or Inflammatory Bowel Diseases
Source: Int J Mol Sci. 2023 May 5;24(9):8279. doi: 10.3390/ijms24098279 (PMC10179007; doi:10.3390/ijms24098279)
Supplement: Supplementary file 1 [file ijms-24-08279-s001.zip › ijms-2241930-supplementary.pdf]

Supplementary Figure S1. Flow-chart showing available saliva samples in the Fin-HIT cohort, selection process, and categorization of saliva samples used in this study.

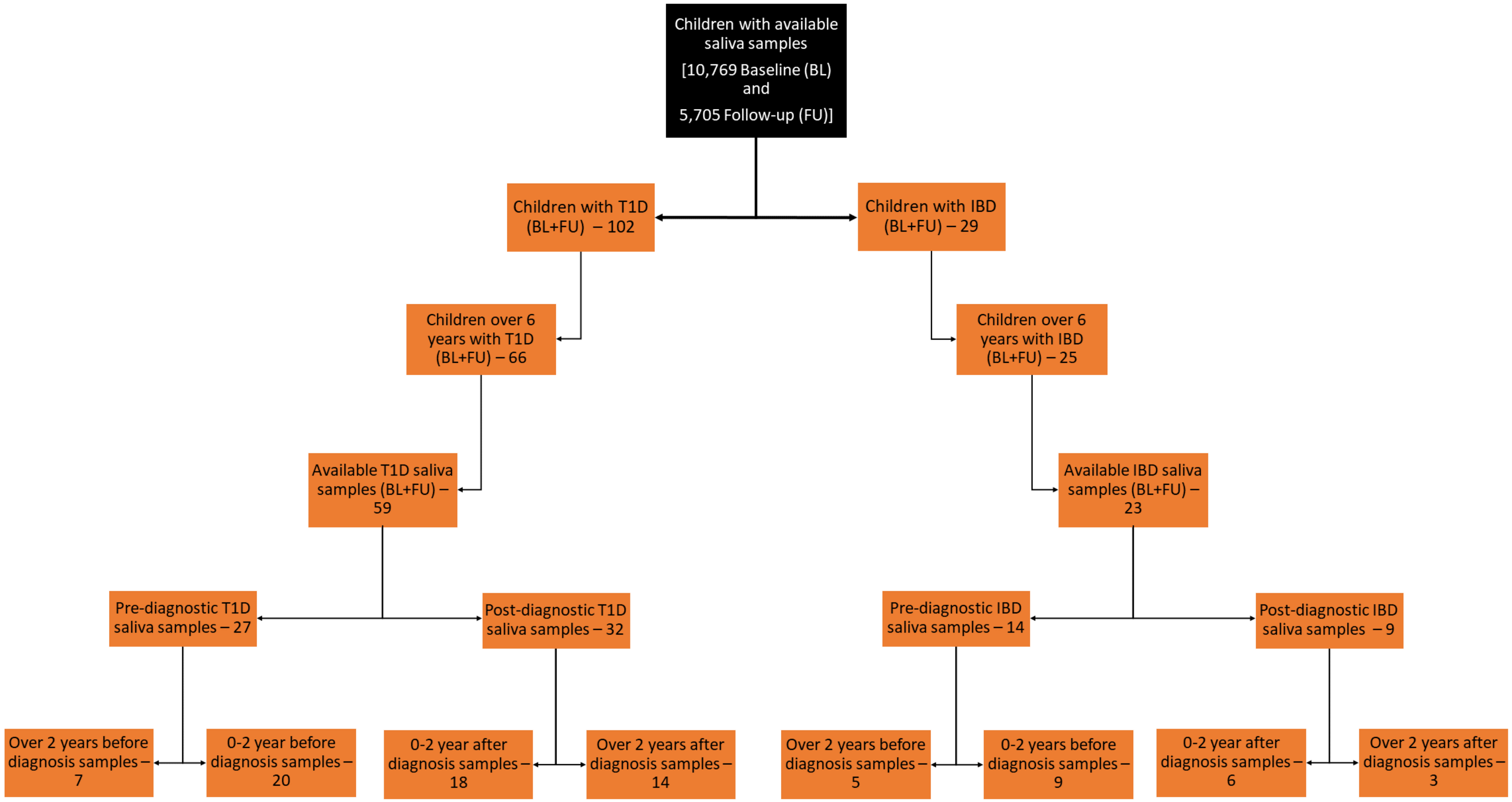

Supplementary Figure S2. PWY-5677 metabolic pathway fermenting succinate to butyrate, which was lower in the saliva of children who developed type 1 diabetes (T1D) within 0-2 years before diagnosis (n=20) than in all controls (n=81). Reprinted from MetaCyc (https://metacyc.org/META/NEW-IMAGE?object=PWY-5677&redirect=T).

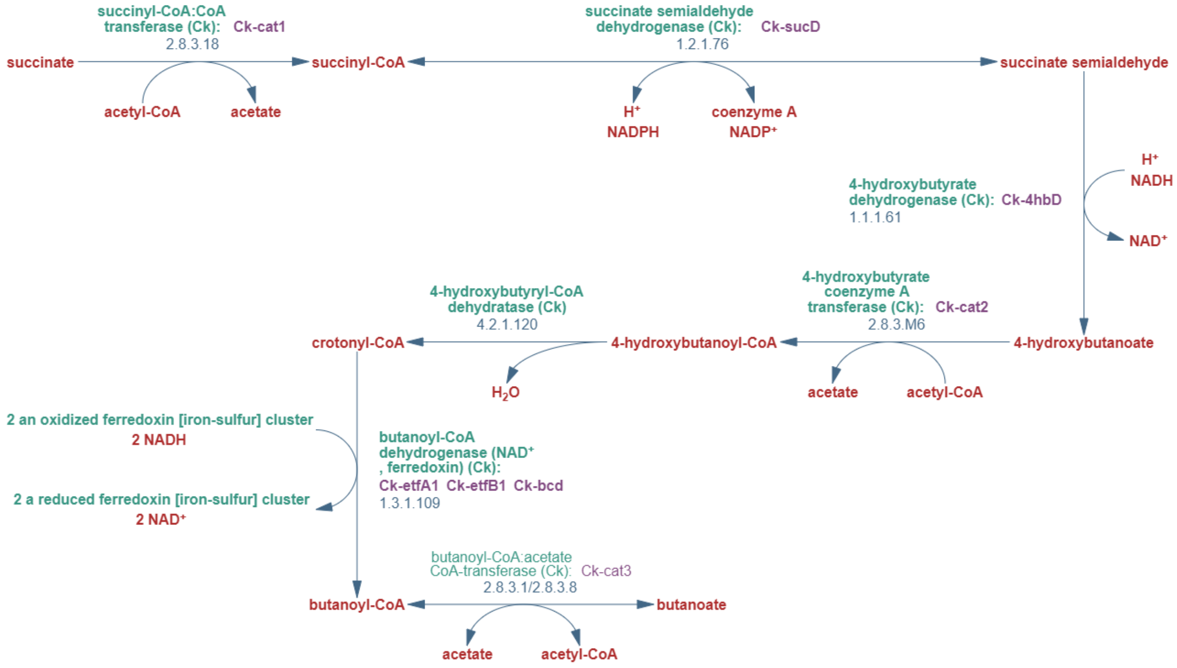

Supplementary Figure S3. Altered metabolic pathways (crude *p*-value ≤ 0.05) within 0-2 years before T1D diagnosis (n=20) when compared with all controls (n=81). PWY-5677 pathway marked in red was the only pathway significant after *p*-value correction.

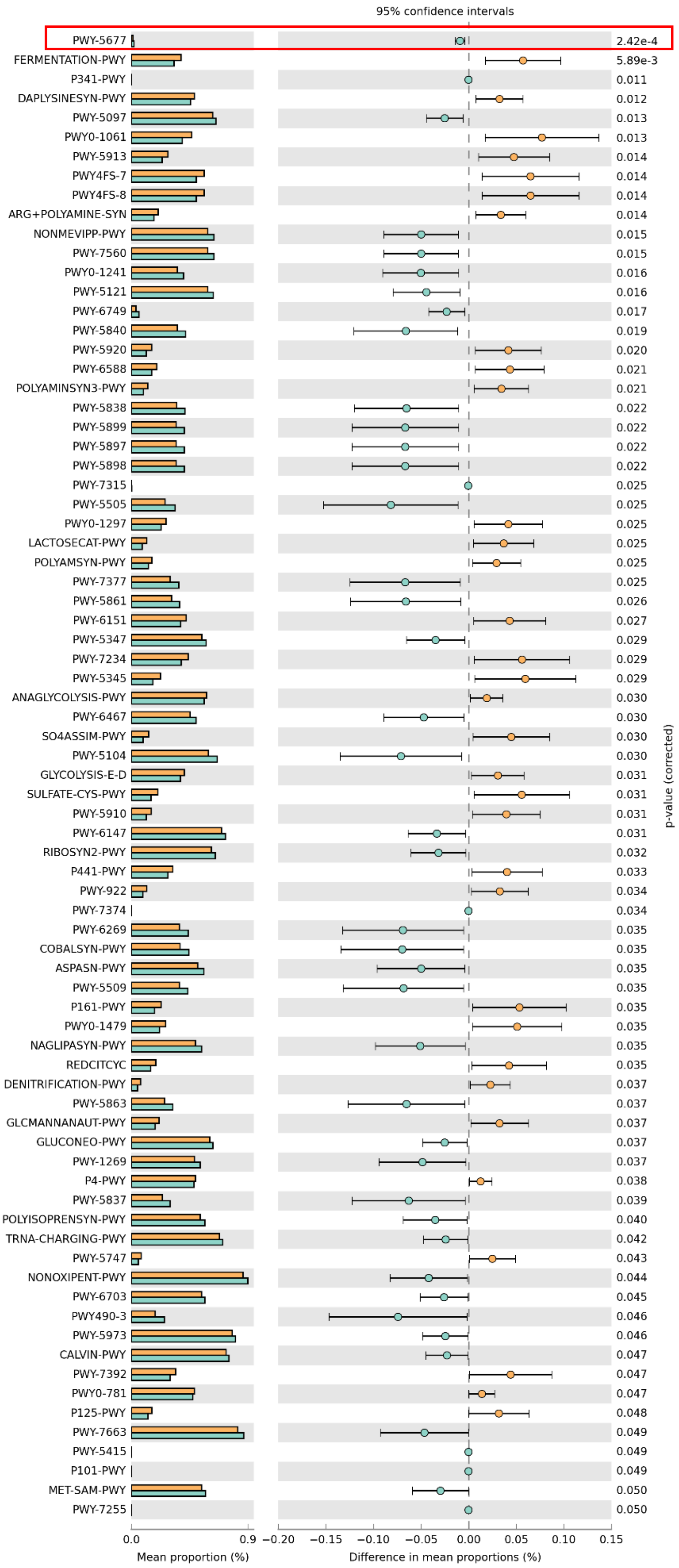

Supplementary Figure S4. Altered metabolic pathways (crude *p*-value ≤ 0.05) for A) all pre-diagnostic IBD samples (n=14; in red) when compared with all controls (n=81; in grey). B) all pre-diagnostic T1D samples (n=27; in green) when compared with all controls (n=81; in grey). None of these pathways remained significant after *p*-value correction.

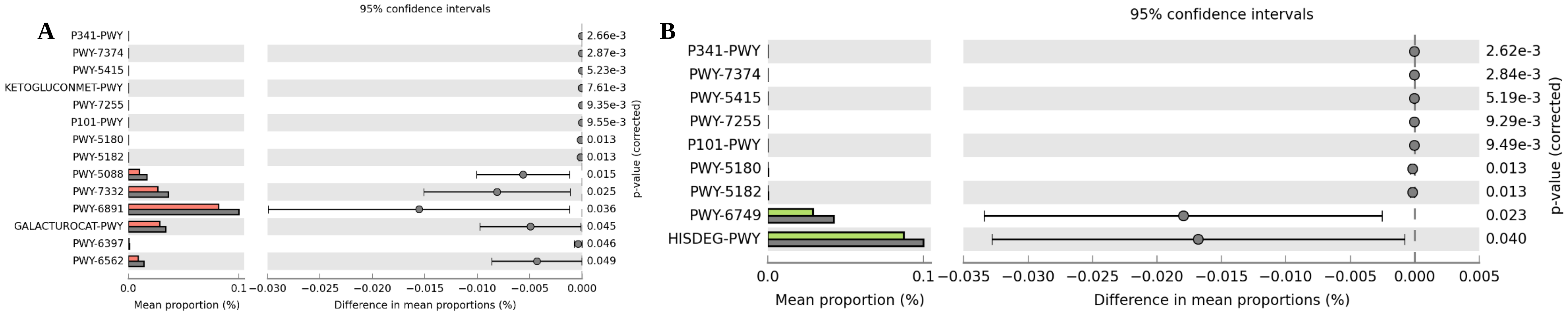

Supplementary Table S1. Comparison of beta-diversity using Permutational Multivariate Analysis of Variance (PERMANOVA) between 1) All pre-T1D and post-T1D samples; 2) All pre-IBD and post-IBD samples; and (3) pre- and post-diagnostic samples from the same individuals with either T1D or IBD (i.e. related samples).

	Comparison of Beta-diversity between Saliva Samples	Sums Of Squares	F statistics	R^2	p-value	p-value (corrected)
1	Pre-T1D vs. Post-T1D	0.017	0.21	0.0035	0.96	0.96
2	Pre-IBD vs. Post-IBD	0.103	1.30	0.0581	0.31	0.52
3	Pre- vs. Post-diagnostic samples from the same individuals with T1D/IBD	0.032	0.33	0.02	0.87	0.87
